# Cataract Preventive Role of Isolated Phytoconstituents: Findings from a Decade of Research

**DOI:** 10.3390/nu10111580

**Published:** 2018-10-26

**Authors:** Vuanghao Lim, Edward Schneider, Hongli Wu, Iok-Hou Pang

**Affiliations:** 1 Integrative Medicine Cluster, Advanced Medical and Dental Institute, Universiti Sains Malaysia, Bertam, Kepala Batas, Penang 13200, Malaysia; vlim@usm.my; 2 Botanical Research Institute of Texas (BRIT), 1700 University Drive, Fort Worth, TX 76107-3400, USA; eschneider@brit.org; 3 Department of Pharmaceutical Sciences, System College of Pharmacy, University of North Texas Health Science Center, Fort Worth, TX 76107, USA; 4 North Texas Eye Research Institute, University of North Texas Health Science Center, Fort Worth, TX 76107, USA

**Keywords:** cataract, phytoconstituents, lens, preclinical models, drug discovery

## Abstract

Cataract is an eye disease with clouding of the eye lens leading to disrupted vision, which often develops slowly and causes blurriness of the eyesight. Although the restoration of the vision in people with cataract is conducted through surgery, the costs and risks remain an issue. Botanical drugs have been evaluated for their potential efficacies in reducing cataract formation decades ago and major active phytoconstituents were isolated from the plant extracts. The aim of this review is to find effective phytoconstituents in cataract treatments in vitro, ex vivo, and in vivo. A literature search was synthesized from the databases of Pubmed, Science Direct, Google Scholar, Web of Science, and Scopus using different combinations of keywords. Selection of all manuscripts were based on inclusion and exclusion criteria together with analysis of publication year, plant species, isolated phytoconstituents, and evaluated cataract activities. Scientists have focused their attention not only for anti-cataract activity in vitro, but also in ex vivo and in vivo from the review of active phytoconstituents in medicinal plants. In our present review, we identified 58 active phytoconstituents with strong anti-cataract effects at in vitro and ex vivo with lack of in vivo studies. Considering the benefits of anti-cataract activities require critical evaluation, more in vivo and clinical trials need to be conducted to increase our understanding on the possible mechanisms of action and the therapeutic effects.

## 1. Introduction

The ocular lens is located at the anterior segment of the eye that, together with the cornea, provides the refractive power of the eye. The mature lens is composed of a core of primary lens fiber cells, layers of secondary lens fiber cells, and one layer of anterior lens epithelial cells, which covers the anterior surface of the lens [1]. The major function of the lens is to maintain transparency so that the light can be properly focused on the retina. Unfortunately, the delicate balance required for lens transparency can be easily disturbed by oxidative stress, aging, and UV radiation, and cataracts develop as a result [1].

Cataracts are the most common cause of vision loss in people over the age of 40 and are the leading cause of blindness in the world [2]. Cataracts are defined as lens opacification that prevents a sharply defined image from reaching the retina. As a result, cataract patients have clouded, blurred, or dim visions, which significantly affect their daily life. According to a report from the World Health Organization, nearly 40 million people are blind worldwide, almost half of them are due to cataract [3]. Although cataract-related vision loss can be corrected by replacement with synthetic lenses, cataract surgery is a costly procedure and may develop complications like infectious endophthalmitis, posterior capsule rupture during surgery, post-operative macular edema, and posterior capsule opacity (also called posterior capsule opacification). In developing countries, many cataract patients cannot have their vision restored due to financial concerns or lack of medical resources. Therefore, identifying a safe compound that can reduce the incidence or delay the onset of cataract is an important step in finding new treatments for cataract.

There have been many compounds evaluated for their potential efficacies in reducing cataract formation. In this article, we focus on active ingredients derived from plants. Phytoconstituents are a trove of often structurally complicated compounds with interesting biological functions. They themselves or their derivatives have always been important sources of pharmacologically active agents. To provide a comprehensive review of potentially useful anti-cataract phytoconstituents, we searched, selected, and extracted the appropriate information from published literature according to the following procedures. We feel that, by listing the comprehensive collection of phytocontituents in one place, this manuscript serves as an overview and perhaps an inspiration to prompt additional studies in this important research area. Collaborative efforts between phytochemists and cataract researchers are promisingly fruitful.

## 2. Materials and Methods

### 2.1. Literature Search

Literature search of articles published from January 2008 to December 2017 was performed. We searched the databases of Pubmed, Science direct, Google Scholar, Web of Science and Scopus using different combinations of keywords: lens epithelial cells, sodium selenite-, ultraviolet radiation-, steroid induced, oxygen-, H_2_O_2_-induced opacity/cataract, congenital/juvenile cataract, transgenic/knockout mice with cataract, diabetic cataract, spontaneous cataract, isolated phytoconstituents, medicinal plants.

### 2.2. Study Selection

**Figure 1:**
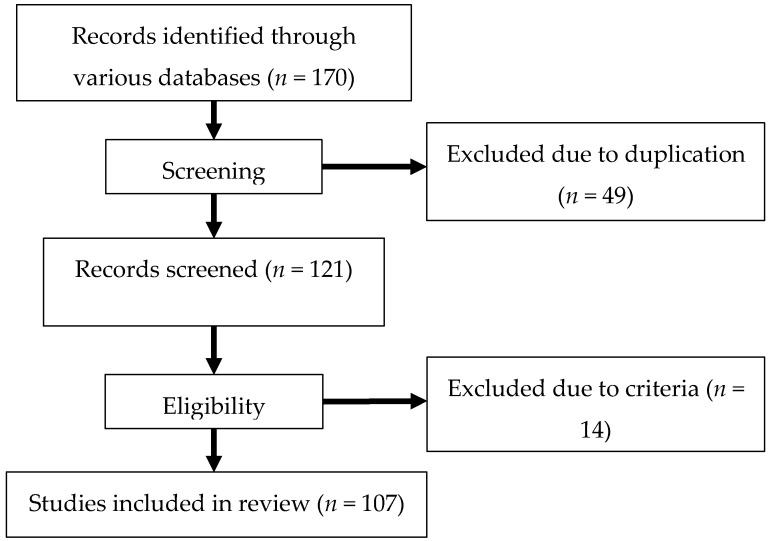
Flow chart of review process in article selection.

The selection of the manuscripts was based on the following inclusion criteria: isolation of phytoconstituents from plants, and selection of phytoconstituent(s) with the most potent anti-cataractogenesis activities. Exclusion of manuscripts from this review involves synthesized/commercialized compounds, phytoconstituents screening without isolated compounds, activities with extracts only and isolated phytoconstituents without names or articles that did not meet the inclusion criteria. The selection process is summarized in Figure 1.

### 2.3. Data Extraction

All the selected manuscripts were analyzed for year of publication, plant species, family, part of plant, solvent extraction, isolation method, isolated phytoconstituents, anti-cataract activities (in vitro, ex vivo or in vivo), route of administration (in vivo), dose or concentration for IC_50_, treatment duration (in vivo) and isolated phytoconstituent(s) with the strongest activity(s), as well as their structural formula. The extracted data are presented in Table 1 and Table 2 throughout this article.

## 3. Results

### 3.1. Experimental Cataract Models

There are a large number of in vitro and in vivo models that mimic certain aspects of the pathophysiological features of human cataracts. They have been used to demonstrate the potential therapeutic effects of phytochemicals. In this section, we describe the most commonly used models in order to aid the understanding and appraisal of results. During our literature search, most of the phytochemicals were tested in in vitro or ex vivo models only. Only a dozen or so were assessed in in vivo cataract models. Nevertheless, for completion’s sake, we list both in vitro and in vivo models. In vitro models discussed are hydrogen peroxide (H_2_O_2_)-, xylose-, galactose-induced lens opacity, aldose reductase (AR) activity assay, and advanced glycation end products (AGE) formation. In vivo models include sodium selenite-, ultraviolet (UV) radiation-, and steroid-induced cataracts. These models have been widely used to study the mechanisms of cataract and serve as the screening platform of anti-cataract therapies with the long-term goal to treat cataract in humans.

### 3.2. In Vitro Models

#### 3.2.1. Oxidative Stress Model

##### H_2_O_2_-Induced Cataract

It is widely accepted that oxidative stress is the major factor for the development of cataracts. Hydrogen peroxide (H_2_O_2_) is the major reactive oxygen species (ROS). H_2_O_2_ is mainly generated in vivo by the detoxification of superoxide (O_2_^−^) radical by superoxide dismutase (SOD) through the dismutation reaction [4,5]. Alternatively, H_2_O_2_ can be produced by a number of oxidase enzymes including monoamine oxidases and peroxisomal pathway for β-oxidation of fatty acids. In the lens, H_2_O_2_ can also be generated by the photochemical reaction [4]. Most human tissues, including the lens, are exposed to some level of H_2_O_2_, with the mitochondria being the major site for production. Previous studies have shown the strong association between H_2_O_2_ overproduction and cataract development. Cataract patients had elevated H_2_O_2_ in both the aqueous body and lens ranging from seven- to 30-fold higher than normal [5]. Lens organ ex vivo culture with H_2_O_2_ in the medium is a common experimental model of cataract. This type of cataract is characterized by loss of GSH and increased protein oxidation. To establish the model, rat or porcine lenses are dissected and cultured in TC-199 medium containing 200 to 1000 μM H_2_O_2_ with final osmolarity of 298 ± 2 mOsm/L. The lens are usually harvested after 24 to 96 h to induce cataracts [6,7].

#### 3.2.2. Diabetic Cataract

Cataract is a major cause of visual impairment in patient with diabetes mellitus. Both clinical and basic research studies have indicated the strong association between diabetes and cataract formation [8,9]. The molecular mechanisms that may be involved in diabetic cataract include polyol pathway flux, increased formation of AGEs, osmotic stress, and elevated oxidative stress.

##### Aldose Reductase (AR) Activity

The polyol pathway, also known as sorbitol-AR pathway, is a two-step process that converts glucose to fructose. AR, the first and rate-limiting enzyme in the pathway, reduces glucose to sorbitol using nicotinamide adenine dinucleotide phosphate (NADPH) as a cofactor. Sorbitol is then converted to fructose by sorbitol dehydrogenase (SDH) [10]. As a sugar alcohol, sorbitol does not diffuse across cell membranes readily. When accumulating intracellularly, it produces osmotic stress on cells by driving water into the lens that may eventually cause diabetic cataract. Therefore, one of the possibilities to prevent the onset of diabetic cataract is to use AR inhibitor (ARI) [11]. The in vitro ARI assay was used to evaluate if the compound can inhibit the polyol pathway. Briefly, the reaction mixture contains 50 μM potassium phosphate buffer pH 6.2, 0.4 mM lithium sulfate, 5 μM 2-mercaptoethanol, 10 μM DL-glyceraldehyde, 0.1 μM NADPH, and freshly-prepared AR enzyme. The reaction is initiated by the addition of NADPH at 37 °C. The AR activity is determined indirectly by a spectrophotometer that measures NADPH absorption [10].

##### Xylose-Induced Lens Opacity

Another in vitro diabetic cataract model is xylose-induced lens opacity. Glucose, galactose, and xylose are all known to induce cataract. Among these three sugars, xylose is the most effective molecule in producing cataracts due to the fact that it is the preferred substrate of AR in the lens. Kinoshita and colleagues first established xylose-induced lens in 1974 [12]. They cultured rat lenses in 4 mL of medium containing 30 mM xylose for six days. They observed a progressive development of lens opacity accompanied by increased osmotic stress and lens swelling [12].

##### Galactose-Induced Lens Opacity

Compared with glucose, galactose has higher affinity with AR and its reduction product galactitol is more difficult to be metabolized by sorbitol dehydrogenase than sorbitol. Therefore, high galactose is more likely to induce sugar cataract than high glucose itself [13]. There are several methods available to establish galactosemic cataract. For example, rat galactosemic cataract can be induced by 30% or 50% galactose diet. Glactose-induced lens opacity can also be achieved by daily intraperitoneal injection of 30–50% galactose solution or daily retrobulbar injection of 20% galactose solution. Another cost efficient way to induce galactosemic cataract is to feed rat 10% galactose solution for 18 days. For in vitro lens culture, 30 mM galactose is added in the culture medium for 72 h incubation [13].

##### Formation of Advanced Glycation End (AGE) Products

Another important factor that is involved with the pathogenesis of diabetic cataract is the formation of AGEs. In diabetic patients with cataract, the elevated glucose starts forming covalent adducts with the lens proteins through a non-enzymatic process called glycation [14,15]. This process is known as one of the most important forms of post-translational modification of proteins under hyperglycemic conditions. Many studies have shown that protein glycation-induced AGEs play a pivotal role in diabetic cataract formation. Therefore, AGE formation assay is used to examine the potential anti-cataract potential of tested compounds. To determine the amount of AGEs, a reaction mixture containing 10 mg/mL of bovine serum albumin and 0.5 M fructose and glucose are mixed with tested compounds. After 15 days of incubation, the fluorescent intensity is measured using a spectrofluorometric detector with an excitation wavelength of 350 nm and an emission wavelength of 450 nm [16,17].

### 3.3. In Vivo Models

Commonly used in vivo models represent specific pathogenesis aspects of human cataract. For example, the diabetic cataract rodent model focuses on mechanisms involved in diabetes-related cataract; selenite-induced cataract addresses oxidative damage-induced cataract; the UV- and steroid-induced models represent their respective associated pathological changes. In various studies, drug effects in these in vivo models correlate well with the pharmacodynamics properties shown in appropriate in vitro models. 

#### 3.3.1. Diabetic Cataract

The in vivo diabetic cataract model can be established by using streptozotocin (STZ). After intraperitoneal (i.p.) or intravenous (i.v.) injection, STZ enters the pancreatic β-cell through the glucose transporter 2 transporter (Glut-2) resulting in hyperglycemia [16]. Moreover, STZ is also a source of free radicals that may lead to DNA oxidative damage and subsequent β-cell death. STZ can be administered as a single high dose (e.g., 160 to 240 mg/kg) or as multiple low doses (e.g., 40 mg/kg for 5 days) [18].

Another commonly used diabetic cataract model is AR transgenic mice. The ubiquitous transgenic and lens-specific AR transgenic mice were developed to further prove that polyol accumulation is responsible for diabetic cataract. In both models, sorbitol accumulates in the lens, causing osmotic swelling, and eventually leading to accelerated diabetic cataract formation [10,19].

#### 3.3.2. Selenite-Induced Cataract

Selenite-induced cataract is an effective, rapid, and reproducible model of nuclear cataracts. Selenite cataract is usually produced either by a single dose (19–30 μM/kg body weight) or repeated smaller dosage of sodium selenite (40–50 nmol/g body weight) subcutaneous injection to suckling rat of 10–14 days of age [20]. It has been proposed that selenite treatment leads to altered metabolism in lens epithelium, including loss of small antioxidant molecules such as glutathione (GSH), decreased rate of epithelial cell differentiation, and increased DNA oxidation damage. Such extensive alterations to the epithelium leads to disrupted calcium homeostasis and calcium accumulation in the nucleus of the lens. Increased calcium activates calcium dependent protease m-calpain (calpain II) which results in rapid proteolysis, precipitation of crystallins, and eventually cataract development in rodent lenses [21,22].

#### 3.3.3. UV-Induced Cataract

UV radiation is a major contributor to the pathogenesis of cataract. The strong energy in the UV light can directly cause a DNA lesion in the lens by inducing thymine dimer formation. More importantly, UV can induce cataract formation by the generation of ROS that indirectly induce oxidative damage to DNA by disturbing cell proliferation in the lens epithelium, altering kinetic properties of enzymes in the energy metabolism, increasing insoluble and decreasing soluble protein, and disturbing the sodium potassium balance, leading to aberrant water balance in the lens [23]. It has been widely accepted that cataract formation is related to oxidative stress induced by continued intraocular penetration of UV light and consequent photochemical generation of ROS such as superoxide and singlet oxygen and their oxidant derivatives such as hydrogen peroxide and hydroxyl radical [24]. Sprague-Dawley rats or mice are exposed to 8 kJ/m^2^ UV-B radiation for 15 min to induce cataracts [25].

#### 3.3.4. Steroid-Induced Cataract

As the steroid hormones, glucocorticoids (GCs) have strong anti-inflammatory effects. By binding with the glucocorticoid receptor (GR), GCs have the ability to inhibit all stages of the inflammatory response [26]. Due to its strong anti-inflammatory effects, GCs are widely used in the management of many clinical conditions, including autoimmune disorders, allergies, and asthma, and they also play important roles in chemotherapy and preventing the rejection after solid organ transplantation. However, prolonged use of GCs is associated with the development of posterior subcapsular and nuclear cataracts [26]. The chick embryo has been used to establish an experimental model to study the response of the lens to GCs. When dexamethasone (0.02 μmol/egg) is administered, the lenses of chicken embryos become cataract within 48 h. More recently, the mammalian lens has also been used to establish the steroid-induced cataract models. For example, Brown-Norway rats given a daily 1% prednisolone acetate instillation of a total volume of 1.0 mg/kg or a daily intramuscular injection of 0.8–1.0 mg/kg prednisolone acetate for 10 months successfully induced morphological changes similar to those found in human steroid-induced cataracts [27,28].

### 3.4. Anti-Cataract Phytoconstituents

Based on our literature strategy listed above, the following phytoconstituents are listed in alphabetical order. They have been shown to possess potential anti-cataract efficacy according to the described study models.

#### 3.4.1. 1-*O*-Galloyl-*β*-d-glucose (β-Glucogallin)

Molecular formula: C_13_H_16_O_10_ (332.262 g/mol), Melting point: 214–216 °C.

β-Glucogallin isolated from the aqueous fruit extract of *Emblica officinalis Gaertn.* (emblic, Indian gooseberry) or *Phyllanthus emblica* Linn. (Euphorbeaceae) (gooseberry) [29] shows potent activity against human AR in vitro with an IC_50_ of 17 µM [30]. Treatment with this compound prevented the sorbitol accumulation by 73% (30 µM) in transgenic human AR expressing lenses ex vivo [30]. This result substantiated the in vitro assay using shared substrate glyceraldehyde at IC_50_ of 58 µM. Treatment with β-Glucogallin produced a significant decrease of sorbitol levels in macrophages [31]. Computational molecular docking studies exhibited favorable binding to the active site of between human AR and β-glucogallin. This corroborates the inhibition result of sorbitol production under hyperglycemic conditions in earlier experiments [30].

#### 3.4.2. 1,3-Di-*O*-caffeoylquinic Acid

Molecular formula: C_25_H_24_O_12_ (516.45 g/mol).

1,3-di-*O*-caffeoylquinic acid has been isolated from *Artemisia iwayomogi* (haninjin) and *Xanthium strumarium* (rough cocklebur) as inhibitor for rat lens AR (RLAR), recombinant human AR (RHAR) and advanced glycation end-product (AGE) inhibitory activities. The compound inhibited RLAR with IC_50_ values of 0.22–1.90 µM [32,33]. This result was supported by inhibition of RHAR at IC_50_ of 0.81 µM. In AGE inhibitory activity, 1,3-di-*O*-caffeoylquinic acid suppressed at IC_50_ of 24.85 µM [33].

#### 3.4.3. 1,5-Di-hydroxy-1,5-di-[(*E*)-3-(4-hydroxyphenyl)-2-propenoic]-3-pentanonyl Ester (DHDP)

Molecular formula: C_23_H_22_O_9_ (442.41 g/mol).

A novel polyphenolic inhibitor of AR, DHDP was isolated from *Lysimachia christinae* (gold coin grass, jinqiancao) using AR affinity-based ultrafiltration-HPLC profiling method. The reversible inhibitory activity of RHAR was recorded at IC_50_ value of 194.7 µM with sorbitol content of 1002.3 µg/g of lens weight. The effect of DHDP was further investigated in in silico using computer simulation of binding by molecular docking. DHDP was predicted to block the AR active site by binding and preventing the formation of product [34].

#### 3.4.4. 1,5-Di-*O*-caffeoylquinic Acid

Molecular formula: C_24_H_24_O_11_ (488.44 g/mol).

A new method of enzyme assay-guided high-performance liquid chromatography microfractionation and elution-extrusion counter-current chromatography of roots ethanolic extract of *Nardostachys chinensis* (spikenard) afforded six secondary metabolites with 1,5-di-*O*-caffeoylquinic acid as the most potent inhibitor against RLAR activity (IC_50_ = 2.98 µM). The compound was reported as the first time isolated from the plant [35].

#### 3.4.5. 1,3,6-Trihydroxy-2-methoxymethylanthraquinone

Molecular formula: C_16_H_12_O_6_ (300.26 g/mol).

Bioassay-guided fractionation of *Knoxia valerianoides* (hongdaji) methanolic root extract afforded eight secondary metabolites with 1,3,6-trihydroxy-2-methoxymethylanthraquinone showing the highest inhibition against AGE formation at IC_50_ value of 52.7 µM. The same phytoconstituent also exhibited strong inhibitory activity against RLAR with IC_50_ value of 3.0 µM [36].

#### 3.4.6. 1,2,3,6-Tetra-*O*-galloyl-*β*-d-glucose

Molecular formula: C_34_H_28_O_22_ (788.57 g/mol).

1,2,3,6-tetra-*O*-galloyl-*β*-d-glucose was isolated from the methanolic seeds extract of *Cornus officinalis* (cornus tree, shan zhu yu) after repeated Sephadex column chromatography. Appeared as an off-white amorphous powder [37], 1,2,3,6-tetra-*O*-galloyl-*β*-d-glucose showed the most potent inhibitory activity (IC_50_ = 0.70 µM) compared to other secondary metabolites. In addition, AGE formation was also reduced to IC_50_ value of 1.99 µM. This compound was further evaluated for its inhibitory effect on ex vivo cataractogenesis activity using rat lenses induced with xylose 20 mM. Treatment with 1,2,3,6-tetra-*O*-galloyl-*β*-d-glucose significantly reduced the opacities of the lenses after two days at the concentration of 80 µM [38].

#### 3.4.7. 1,3,5,8-Tetrahydroxyxanthone

Molecular formula: C_13_H_8_O_6_ (260.19 g/mol).

Several xanthones have been isolated from the ethanolic extract of *Swertia mussotii* Franch (yinchen) as inhibitors for RLAR activity. The most potent inhibition was shown by 1,3,5,8-tetrahydroxyxanthone with IC_50_ of 0.0886 µM [39]. The compound appeared in slight yellow powder with 98.6% purity.

#### 3.4.8. 2″,4″-*O*-Diacetylquercitrin

Molecular formula: C_25_H_24_O_13_ (532.11 g/mol), Melting point: 187 °C.

2″,4″-*O*-Diacetylquercitrin was isolated from *Melastoma sanguineum* (red melastome, fox-tongued melatsome) as a yellow amorphous powder. This compound exhibited the strongest inhibition against RLAR and AGE activities among all the isolated phytoconstituents. IC_50_ inhibitory activities for RLAR and AGE were recorded at 0.077 µM and 11.46 µM, respectively. Compared to the positive standards, aminoguanidine (IC_50_ = 965.9 µM, AGE) and 3,3-tetramethyleneglutaric acid (IC_50_ = 28.8 µM, RLAR), 2″,4″-*O*-Diacetylquercitrin inhibited 87 (AGE) and 374 (RLAR) times more efficaciously [40].

#### 3.4.9. 3-Isomangostin

Molecular formula: C_24_H_26_O_6_ (410.46 g/mol), Melting point: 182–183 °C.

Three main constituents of dichloromethane extract from root bark of *Garcinia mangostana* Linn (mangosteen) were isolated from the hexane/methanol fraction. The result of the study indicated that 3-isomangostin possessed the highest RLAR inhibitory activity with at IC_50_ value of 3.28 µM. The presence of cyclization of the prenyl group at the position-two carbon with xanthone derivative enhanced the structure-activity relationship [41].

#### 3.4.10. 3′,4-Dihydroxy-3,5′-dimethoxy-bibenzyl (Gigantol)

Molecular formula: C_16_H_18_O_4_ (274.316 g/mol), Melting point: 135 °C.

Gigantol is a bibenzyl-type phenolic compound presents in most herbs of Orchidaceae family [42]. It has been isolated from the stems of various *Dendrobium* genus such as *Dendrobium aurantiacum* var. *denneanum* (die qiao shi hu) [43,44] and *Dendrobium chrysotoxum* Lindl (fried-egg orchid) [45,46] for anti-cataract activities. As a white solid, gigantol suppresses the damage of rat lenses both in vitro and in vivo in galactose-induced cataractogenesis. The delay in lens turbidity was caused by the inhibition of AR and inducible nitric oxide synthase mRNA expression at an IC_50_ of 239.4 µM (65.7 µg/mL) and 32.0 µM (8.8 µg/mL), respectively [43]. Gigantol isolated from *Dendrobium chrysotoxum* Lindl interpolated into the DNA base pairs in AR gene with a binding constant of 1.85 × 10^3^ L/mol, thus, suppressed the gene expression [46].

#### 3.4.11. 3′,5′-Dimethoxy-(1,1′-biphenyl)-3,4-diol 3-*O*-*β*-d-glucopyranoside

Molecular formula: C_20_H_24_O_9_ (406.42 g/mol).

The leaves and twigs of *Osteomeles schwerinae* C. K. Schneid. (hu xi xiao shi ji) were examined for their possible inhibitory activity on RLAR. Four secondary metabolites have been isolated from the CHCl_3_-MeOH fraction of the EtOH extract and found that 3′,5′-dimethoxy-(1,1′-biphenyl)-3,4-diol 3-*O*-*β*-d-glucopyranoside to be the most potent inhibitor against RLAR activity at IC_50_ value of 3.8 µM. The phytoconstituent was obtained as a brownish powder [47].

#### 3.4.12. 3,5-Di-*O*-caffeoylquinic Acid

Molecular formula: C_25_H_24_O_12_ (516.45 g/mol), Melting point: 184–187 °C.

Methanolic extract of the stems and leaves of *Erigeron annuus* (annual fleabane, daisy fleabane) afforded 16 secondary metabolites. 3,5-di-*O*-caffeoylquinic acid appeared as pale-yellow powder and isolated from the ethyl acetate-soluble fraction after repeated column chromatography [48,49,50]. The same constituent was also isolated from *Aster koraiensis* (Korean starwart) [51], *Xanthium strumarium* (clotbur, common cocklebur) [33], *Artemisia iwayomogi* (haninjin) [32] and *Artemisia montana* [52]. 3,5-di-*O*-caffeoylquinic acid was reported as the most significant inhibitory activities against AGEs, RLAR and ex vivo xylose-induced lens opacity assays from all isolated constituents. It attenuates AGE formation with IC_50_ values ranging from 6 µM to 32 µM, and inhibits RLAR with IC_50_ values of 0.2 to 5 µM. These findings are further substantiated by its ability in inhibition of galactitol accumulation at an IC_50_ of 153 µM [33] and prevention of xylose-induced opacity of lenses at a concentration of 10 µM [48].

#### 3.4.13. 4-*O*-Butylpaeoniflorin and Palbinone

Molecular formula of 4-*O*-butylpaeoniflorin: C_27_H_36_O_11_ (536.22 g/mol), Melting point: 173–175 °C.

Molecular formula of Palbinone: C_22_H_30_O_4_ (359.47 g/mol), Melting point: 254–255 °C.

Both 4-*O*-butylpaeoniflorin and Palbinone were isolated from methanolic extract of the cortex of *Paeonia suffruticosa* (tree peony, mudan, moutan) with highest inhibitory activities of RLAR (palbinone) and AGE (4-*O*-butylpaeoniflorin) compared to other isolated phytoconstituents [53]. Palbinone appeared as red needles with [α]_D_ −223.8° (CHCl_3_) and absorbed UV at 237 (log ɛ: 3.2) and 387 nm (log ɛ: 3.0) [54]. Isolated from the butanol fraction. 4-*O*-butylpaeoniflorin was found as an optically active white foam, ${\left[\alpha \right]}_{D}^{25}$–7.8 (*c* 0.14, MeOH) and later confirmed as an extraction artifact after HPLC analysis. Palbinone inhibits RLAR at an IC_50_ value of 11.4 µM. It was suggested that the absence of ring E, side chain of ring D together with double bonds and a conjugated carbonyl group on the ring D played the inhibitory properties. Unlike palbinone, 4-*O*-butylpaeoniflorin inhibited (IC_50_ = 10.8 µM) for AGE activity. The chemical moiety of hydroxy groups in the benzoyl connected to the sugar unit complement the activity [53].

#### 3.4.14. 4,5-Di-*O*-trans-caffeoyl-d-quinic Acid

Molecular formula: C_25_H_24_O_12_ (516.45 g/mol).

Caffeoylquinic acid analog, 4,5-Di-*O*-trans-caffeoyl-d-quinic acid isolated from *Hydrangea macrophylla* var. *thunbergii* (bigleaf hydrangea) and *Ilex paraguariensis* (Yerba mate) showed the strongest inhibitory activity against RLAR at IC_50_ value of 0.29 µM [55]. Inhibitory effect of quinic acid with two caffeoyl groups assisted the potency.

#### 3.4.15. 5-*O*-Feruloly Quinic Acid

Molecular formula: C_17_H_20_O_9_ (368.33 g/mol)

Bioassay-guided isolation of root methanolic extract of *Aralia continentalis* Kitag. (dong bei tu dang gui) produced 18 secondary metabolites. 5-*O*-Feruloly quinic acid was isolated from the ethyl acetate fraction as an amorphous white powder. It had a highest inhibitory activity of RLAR at IC_50_ value of 14.2 µM among all other phytoconstituents [56].

#### 3.4.16. 5,7,4′ Trihydroxyisoflavone (Genistein)

Molecular formula: C_15_H_10_O_5_ (270.24 g/mol), Melting point: 297–298 °C.

Genistein appears as colorless plates and isolated from the roots of *Pueraria lobata* (kudzu, Japanese arrowroot) [57,58] and stem bark of *Maackia amurensis* (Amur maackia) [59]. Both plants are native to Eastern Asia and used as traditional medicine in China, Korea, and Japan. Genistein shows a significant dose-dependent inhibition on RLAR activity (IC_50_ = 9.48 µM) compared to the positive control, TMG (3,3-tetramethyleneglutaric acid) (IC_50_ = 28.70 µM). Nevertheless, IC_50_ was recorded higher at 57.1 µM for the same activity compared to quercetin IC_50_ = 10.1 µM) [59]. In an ex vivo lens opacity study genistein suppressed xylose-induced lens opacity at 5 µg/mL (18.5 µM). Further analysis with human lens epithelia cells (LECs; HLE-B3 cells) found that the expression of TGF-β2, αβ-crystallin, and fibronectin mRNAs were reduced, suggesting genistein is protective against lens opacity with antioxidative effects [60]. It is proposed that the chemical moiety with free hydroxyl group at C-7 of genistein attributes to the inhibitory of AR [59].

#### 3.4.17. 20(*S*)-Ginsenoside Rh2

Molecular formula: C_36_H_62_O_8_ (622.87 g/mol).

20(*S*)-Ginsenoside Rh2 is classified under triterpene glycosides and isolated from the root of *Panax ginseng* C. A. Meyer, (ginseng). It has been used traditionally in East Asia for many years ago with many main active constituents, ginsenosides have been isolated. In RHAR inhibitory activity, 20(*S*)-Ginsenoside Rh2 showed the most potent inhibitor with an IC_50_ of 147.4 µM among all other isolated ginsenosides. It was suggested that the moiety of hydroxyl group at the carbon-20 enhanced the AR activity relationship [61].

#### 3.4.18. Acteoside

Molecular formula: C_29_H_36_O_15_ (624.58 g/mol), Melting point: 143–146 °C.

Isolated as yellowish amorphous powder from methanolic extract of *Abeliophyllum distichum* (forsythia) and leaves and stem ethanolic extracts of *Brandisia hancei* (laijiangteng), acteoside showed the highest RLAR inhibitory activities at IC_50_ values ranging 0.83 µM and 1.39 µM compared to four other isolated phenolic glycosides from each plant, respectively. The isolation was conducted by high-speed counter current chromatography using a solvent system of ethyl acetate:n-butanol:water [62]. Isolated acteoside from *Brandisia hancei* showed potent AGE inhibitory activity with an IC_50_ value of 5.11 µM [63].

#### 3.4.19. Basilicumin [7-(3-hydroxypropyl)-3-methyl-8-*β*-*O*-d-glucoside-2H-chromen-2-one]

Molecular formula: C_19_H_24_O_9_ (396.38 g/mol).

Basilicumin was isolated from *Ocimum basilicum* (basil). It exhibits potent inhibitory activity against AR (AKR1B1) and aldehyde reductase (AKR1A1) compared to the second phytoconstituent isolated, ocimunone. Basilicumin inhibited AKR1A1 at IC_50_ value of 0.78 µM and 2.1 µM for AKR1B1 activity. It was suggested that coumarin and glucose scaffold in basilicumin moiety enhance the activity [64].

#### 3.4.20. Caffeic Acid

Molecular formula: C_9_H_8_O_4_ (180.16 g/mol), Melting point: 223–225 °C.

Caffeic acid appears as white amorphous powder and classified as a hydroxycinnamic acid [65]. In an attempt to find potential cataractogenesis inhibitors from plants, caffeic acid has been isolated from a few plants with potential activity. Isolation of caffeic acid for RLAR activity has been shown from methanolic extract of *Dipsacus asper* (xuduan), *Erigeron annuus* (L.) Pers., and *Phellinus linteus* (black hoof mushroom, meshima, song gen, meshimakobu, sanghwang) with the highest activity among all isolated secondary metabolites at IC_50_ values of 16.7 µM to 55 µM. Comparable results were observed for RHAR (IC_50_ = 55 to 210 µM) and AGE activities (IC_50_ = 7.6 µM) [66,67,68]. Interestingly, no inhibitory effects were observed for caffeic acid isolated from *Perilla frutescens* L. [69] and *Prunella vulgaris* L. [70].

#### 3.4.21. Canangafruiticoside E

Molecular formula: C_25_H_32_O_12_ (524.51 g/mol).

The repeated column chromatography of methanolic flower bud extract of *Cananga odorata* Hook. F. and Thomson generated 25 secondary metabolites and they were tested for RLAR inhibitory activity. The result of the study indicated that among the isolated constituents, canangafruiticoside E possessed the highest activity (IC_50_ = 0.8 µM) [71].

#### 3.4.22. Capsofulvesin A [((2*S*)-l-*O*-(6Z,9Z,12Z,15Zoctadecatetraenoyl)-2-*O*-(4Z,10Z,13Zhexadecatetraenoyl)-3-*O*-*β*-d-galactopyranosyl Glycerol)]

Molecular formula: C_45_H_72_O_10_ (773.04 g/mol).

The isolation of Capsofulvesin A from ethanolic extract of *Capsosiphon fulvescens* (one of the green algae) showed the strongest RLAR inhibitory activity among all other secondary metabolites, albeit moderate activity at IC_50_ value of 52.5 µM. However, the constituent did not show any inhibition against AGE activity [72].

#### 3.4.23. Caryatin-3′ methyl ether-7-*O*-*β*-d-glucoside

Molecular formula: C_24_H_26_O_12_ (506.14 g/mol).

The bark of the pecan tree (*Carya illinoinensis* (Wangenh) K. Koch) has shown good inhibition of AR activity with few compounds have been isolated. Among them, caryatin-3′ methyl ether-7-*O*-*β*-d-glucoside exhibits the most powerful activity in suppressing the lens AR levels in diabetic cataract rats [73]. The catechol moiety on the B ring of caryatin-3′ methyl ether-7-*O*-*β*-d-glucoside was suggested to inhibit AR in comparison to the activity of other compounds isolated. In addition, the potent AR activity was also supported by the presence of neighboring *O*-methyl group in phenolics and an OH group at C-4′ [73,74,75]. Caryatin-3′ methyl ether-7-*O*-*β*-d-glucoside is physically yellow amorphous powder with UV (MeOH) λ_max_ absorption at 350, 330, and 260 nm [73].

#### 3.4.24. C-Phycocyanin (C-PC)

Molecular formula: C_33_H_38_N_4_O_6_ (586.67 g/mol).

C-Phycocyanin (C-PC), a prominent phytoconstituent found in the stromal surface of thylakoid membranes of *Spirulina platensis* (a blue-green algae) is a biliprotein that functions to capture light energy to chlorophyll A [76,77,78]. As C-PC is miscible in water but not alcohol and esters, most of the isolations of C-PC use water extraction method [79]. C-PC attenuates selenite-induced cataractogenesis both in vitro and in vivo rat model [78,80]. In vitro study showed C-PC recorded low degree of opacification at 200 µg C-PC with 100 µM sodium selenite [78]. The purified C-PC was active toward the in vivo selenite mediated cataractogenesis showing only slight opacification at 200 mg/kg [78]. Same concentration was observed for naphthalene- and galactose-induced cataract rat models [81]. The protective effect of C-PC in these models were proven from the increment of glutathione, soluble proteins, and water content levels of the lens [79]. Histology study indicated the protection of the lens from oxidative damage. Restoration of lenticular micro-architecture was found with C-PC treated group [77]. C-PC maintains the lens transparency by transcriptional regulation of crystallin, redox genes, and apoptotic cascade mRNA expression [80]. Furthermore, C-PC was suggested to possess protective effects on human LEC by abrogating d-galactose-induced apoptosis through the mitochondrial pathway (p53 and Bcl-2 family protein expression) and unfolded protein response pathway (GRP78 and CHOP expression) [82].

#### 3.4.25. Davallialactone

Molecular formula: C_25_H_20_O_9_ (464.43 g/mol).

Davallialactone was isolated as yellow amorphous powder from the active ethyl acetate fraction of fruiting body of *Phellinus linteus*. Davallialactone possessed the most potent inhibitory against RLAR and RHAR among all the isolated compounds with IC_50_ values of 0.33 µM and 0.56 µM, respectively. The inhibitory activities were nine times (RLAR) and 11 times (RHAR) compared to that of quercetin (IC_50_ = 2.91 µM; RLAR and IC_50_ = 6.27 µM; RHAR) [67].

#### 3.4.26. Delphinidin 3-*O*-*β*-galactopyranoside-3′-*O*-*β*-glucopyranoside

Molecular formula: C_27_H_31_O_17_ (627.52 g/mol).

Anthocyanin delphinidin 3-*O*-*β*-galactopyranoside-3′-*O*-*β*-glucopyranoside was isolated from the methanolic extract of the air-dried fruit pericarp of *Litchi chinensis* Sonn (lychee). This fruit is a tropical and subtropical edible fruit native to Southeast Asia. Delphinidin 3-*O*-*β*-galactopyranoside-3′-*O*-*β*-glucopyranoside exhibits the most significant inhibitory activity in RLAR assay with an IC_50_ value of 0.23 µg/mL (0.37 µM) compared to the positive control, tetramethylene glutaric acid (IC_50_ = 0.48 µg/mL) [83].

#### 3.4.27. Desmethylanhydroicaritin

Molecular formula: C_20_H_18_O_6_ (356.36 g/mol), Melting point: 220–222 °C.

The isolation of repeated chromatography of the CH_2_Cl_2_ fraction over a silica-gel column and Sephadex LH20 from root methanolic extract of *Sophora flavescens* (kushen) afforded desmethylanhydroicaritin. Desmethylanhydroicaritin exerted remarkable inhibitory activity of RLAR with IC_50_ value of 0.95 µM. Comparable results were observed in RHAR and AGE inhibitions where IC_50_ values were observed at 0.45 µM and 294.6 µM, respectively. The presence of prenyl and lavandulyl groups enhanced the RLAR and RHAR inhibitory activities. The 3-hydroxyl group at prenylated flavonoids was suggested for the structural contribution for inhibition of AGE formation [84].

#### 3.4.28. Ellagic Acid

Molecular formula: C_14_H_6_O_8_ (302.19 g/mol), Melting point: ≥350 °C.

During a search for possible cataractogenesis activities for isolated ellagic acid, three plants from Korea were found with most potent inhibitions. Ellagic acid isolated from *Phellinus linteus*, *Geranium thunbergii* (Thunberg’s geranium), and *Syzygium cumini* (L.) Skeels (jambolan, Java plum, black plum) inhibits RLAR activity with IC_50_ value ranging from 0.12 µM to 6.9 µM [67,85,86,87]. The compound was also effective in the inhibition of AGE formation (IC_50_ = 26.0 µM). In RHAR assay, the activity of ellagic acid (IC_50_ = 1.37 µM) from *Phellinus linteus* was more potent than that of quercetin (IC_50_ = 6.27 µM) [67]. This was substantiated by its inhibition (42.5%) of galactitol accumulation in rat lenses incubated in high glucose with 485.6 µg/lens wet weight [85].

#### 3.4.29. Epiberberine

Molecular formula: C_20_H_18_NO_4_^+^ (336.36 g/mol), Melting point: 187 °C.

The bioassay-guided isolation of the rhizome of *Coptis chinensis* Franch (Chinese goldthread) afforded seven secondary metabolites with epiberberine exhibited the highest inhibitory of RLAR activity. The IC_50_ of the reported value was 100 µM. Conversely, epiberberine showed a comparable result against RHAR with IC_50_ value of 168.1 µM. The chemical moiety of dioxymethylene (ring D) and its oxidized form (ring A) was suggested to enhance the AR inhibitory activities, albeit in moderate effects [88].

#### 3.4.30. Geraniin

Molecular formula: C_41_H_28_O_27_ (952.64 g/mol), Melting point: 360 °C.

The anti-cataract activities of *Nephelium lappaceum* (rambutan) [89] and *Geranium thunbergii* [85] lead to the isolation of geraniin with good yield. Geraniin was isolated from the ethanolic rind extract of *Nephelium lappaceum* as the major bioactive compound. This compound exhibits better AR activity with an IC_50_ of 0.15 µM at approximately 40% higher compared to quercetin IC_50_ = 5.76 µM [89]. Geraniin isolated from *Geranium thunbergii* shows slightly higher concentration of IC_50_ (8.54 µM) in the same activity, however, using rat lens as the source of enzyme [85]. In AGE assay, the activity of geraniin was 96% of inhibition after incubation time of seven days at the concentration of 20 µg/mL (21 µM). Galactitol accumulation in rat lenses incubated with high galactose was inhibited at 39.9% by geraniin with 507.5 µg/lens wet weight (g). It was concluded that geraniin isolated from both plants is a promising agent in the prevention or treatment of diabetic complications.

#### 3.4.31. Hipolon

Molecular formula: C_12_H_12_O_4_ (220.22 g/mol), Melting point: 237.5–238.5 °C.

Three inhibitors have been isolated from ethanolic extract of *Phellinus merrillii* (willow) fruiting body and identified as hispidin, hispolon, and inotilone. Hipolon showed highest inhibition against RLAR activity (IC_50_ = 9.47 µM) among the three suggesting that phenolic chemical moiety enhanced the activity [90].

#### 3.4.32. Hirsutrin

Molecular formula: C_21_H_20_O_12_ (462.40 g/mol), Melting point: 156–157 °C.

Hirsutrin was isolated together with six nonanthocyanin and five anthocyanin compounds from *Zea mays* L. (corn) for anti-cataractogenesis activity. Isolation of hirsutrin was conducted through bioassay-guided fractionation of ethanolic extract from the kernel of *Zea mays* L. using repeated column chromatography from ethyl acetate fraction. Hirsutrin showed the highest inhibitory activity in RLAR with an IC_50_ value of 4.78 µM and inhibitory constant (*K_i_*) at 7.21 × 10^−7^ M from secondary plots of Lineweaver-Burk plots for RHAR assay. Further inhibition by hirsutrin on galactitol formation in rat lens (33.8% inhibition) and erythrocytes (15.7 µM, 32.5% inhibition) supported the efficacy of hirsutrin as the most effective AR inhibitors compared to all isolated compounds [91].

#### 3.4.33. Hopeafuran

Molecular formula: C_28_H_18_O_7_ (466.43 g/mol), Melting point: 131–134 °C.

Hopeafuran, classified under oligostilbenoids was isolated from the bark of *Shorea roxburghii* (white meranti) and exhibits the highest RLAR inhibitory activity compared to other isolated secondary metabolites from the same plant. This phytoconstituent inhibits the AR enzyme at an IC_50_ value of 6.9 µg/mL (14.8 µM) [92].

#### 3.4.34. Hypolaetin 7-*O*-[6‴-*O*-acetyl-*β*-d-allopyranosyl-(1→2)]-6″-*O*-acetyl-*β*-d-glucopyranoside

Molecular formula: C_31_H_34_O_19_ (710.59 g/mol).

Hypolaetin 7-*O*-[6‴-*O*-acetyl-*β*-d-allopyranosyl-(1→2)]-6″-*O*-acetyl-*β*-d-glucopyranoside was isolated from *Sideritis brevibracteata* (Dağ çayı) [93] and appeared as yellow powder [94,95]. This plant is native to Turkey and widely used as an herbal tea in folk medicine. Isolated hypolaetin has shown the most potent inhibitory activity of AR with IC_50_ value of 0.66 µM [93].

#### 3.4.35. Isocampneoside II

Molecular formula: C_29_H_36_O_16_ (640.58 g/mol).

Isocampneoside II is an active phenylethanoid glycoside isolated from acetone-H_2_O (7:3, *v*/*v*) seeds extract of *Paulownia coreana* (kiri, paotong) at room temperature for 72 h. *Paulownia coreana* is long cultivated in Eastern Asia, particularly Korea and has been used traditionally in medicines for certain ailments [66]. A total of nine potential inhibitors have been isolated from this plant, however Isocampneoside II is the most potent inhibitor in anti-cataract activities. This compound significantly and uncompetetively inhibited RHAR activity with an IC_50_ value of 9.72 µM [66].

#### 3.4.36. Isorhamnetin-3-glucoside

Molecular formula: C_22_H_22_O_12_ (478.406 g/mol), Melting point: 168–172 °C.

*Cochlospermum religiosum* (silk-cotton tree, buttercup tree) has been reported to possess anticataract activity [96]. Purification of hot 95% ethanolic leaves extract of *C. religiosum* yielded isorhamnetin-3-glucoside. This bioactive compound was obtained as yellow needles and identified as flavonoids with yellowish orange color in alkali, pink in Mg-HCl and reaction with Fe^3+^ gives olive green color. Isorhamnetin-3-glucoside at the concentration of 25 µg/mL (52 µM) inhibited further formation of vacuoles and opacity on sodium selenite-induced lens opacity of rat pups. The antioxidant property of isorhamnetin-3-glucoside was suggested to complement its anticataract activity [97].

#### 3.4.37. Kaempferol

Molecular formula: C_15_H_10_O_6_ (286.23 g/mol), Melting point: 276–278 °C.

Kaempferol isolated from *Litsea japonica* (Thunb.) Juss. (hamabiwa) showed the most potent against RLAR inhibitory activity with IC_50_ value of 1.10 µM among all phytoconstituents isolated [98]. The same constituent was also isolated from *Agrimonia pilosa* Ledeb (hairy agrimony, hangul), *Allium victorialis* (victory onion), and *Paulownia coreana* with IC_50_ values of 15.2 µM (RLAR) [99], 1.10 µM (RLAR) [100], and 45.58 µM (RHAR) [66], respectively. The cataract prevention was further supported by inhibition of AGE activity at IC_50_ of 36.01 µM from *Allium victorialis* [100].

#### 3.4.38. Kakkalide

Molecular formula: C_28_H_32_O_15_ (608.549 g/mol), Melting point: 251–253 °C.

Kakkalide was isolated from *Viola hondoensis* W. Becker et H Boss (ri ben qiu guo jin cai) for its AR inhibitory activity. This plant is widely distributed in southern Korea and has been used to as traditional medicine in the form of expectorant. AR activity-guided isolation using column chromatography on a silica gel and gel filtration column afforded kakkalide. Kakkalide significant inhibited AR from Sprague-Dawley rat lenses at an IC_50_ of 0.34 µg/mL (0.56 µM), more potent than that of the positive control, tetramethylene glutaric acid (IC_50_ = 0.48 µg/mL) [101].

#### 3.4.39. Lucidumol A [(24*S*)-24,25-Dihydroxylanost-8-ene-3,7-dione]

Molecular formula: C_30_H_48_O_4_ (472.69 g/mol).

Lucidumol A is a new triterpenoid isolated from the ethanolic extract of the fruiting body of *Ganoderma lucidum* (lingzhi mushroom, reishi mushroom) from a thorough fractionation process [102]. Obtained as a white amorphous powder [103], lucidumol A suppressed the strongest AR activity with an IC_50_ of 19.1 µM compared to all other reported isolated secondary metabolites including ganoderic acid Df (IC_50_ = 22.8 µM) [104], ganoderic acid C2 (IC_50_ = 43.8 µM) [105], ganoderol B (IC_50_ = 110.1 µM) [106], and others.

#### 3.4.40. Lupeol

Molecular formula: C_30_H_50_O (426.72 g/mol), Melting point: 120–122 °C.

Lupeol, a pentacyclic triterpenoid has been isolated from the ethanolic flower extract of *Musa* sp. var. Nanjangud rasa bale (banana) [107] and methanolic leaf extract of *Vernonia cinereal* (purple fleabane) [108] with anti-cataractogenesis activities. Repeated silica gel chromatography after fractionation from both plants yielded lupeol as white needles. It inhibits human recombinant AR activity at IC_50_ of 1.53 µg/mL (3.6 µM) [52]. Similar inhibition was observed for AGE with inhibition in the range of 79–82% [107]. The potent activity of lupeol was substantiated with in vivo study using selenite-induced cataract formation in Sprague-Dawley rat pups. Lupeol attenuated the formation of vacuoles and opacity of rat pups lenses at the concentration of 25 µg/g in a dose-dependent manner in selenite-induced cataractogenesis [108].

#### 3.4.41. Luteolin (2-(3,4-dihydroxyphenyl)-5,7-dihydroxy-4-chromomenone)

Molecular formula: C_15_H_10_O_6_ (286.24 g/mol), Melting point: >320 °C.

The potential anti-cataract effect of luteolin is well known [109,110]. As yellow crystalline, luteolin has been isolated from various plants including *Platycodon grandiflorum* (balloon flower, 

Chinese bellflower) [111], *Vitex negundo* (Chinese chastetree, horseshoe vitex) [112], *Artemisia montana* [52], *Perilla frutescens* (L.) (perilla, Korean perilla) [69,113,114] and *Sinocrassula indica* (Chinese crassula) [115]. The selenite-induced oxidative stress treated group with luteolin (isolated from *Vitex negundo*) demonstrated 80% transparency of the lenses with minor cortical vacuolization and opacity suggesting that the anticataractogenic effect was supported by the antioxidant property based on significant decrease in various antioxidant activities tested [112]. In comparison to the isolated luteolin from different botanicals, luteolin from *Platycodon grandiflorum* was identified as the highest inhibition with an IC_50_ of 0.087 µM (RLAR) [111]. The IC_50_ value increases slightly higher to 0.45 µM (*Sinocrassula indica*) in the same activity [116]. However, isolated luteolin from the same species, *Perilla frutescens* (L.) of different parts showed different values in RLAR (seeds, IC_50_ 0.6 µM [113]; IC_50_ 1.89 µM) [114] and RHAR (leaves, IC_50_ 6.34 µM) [69], almost 10 and 3.5 times higher. Luteolin (*Artemisia montana*) was found to suppress RLAR activity at an IC_50_ 0.19 µM [52]. Luteolin from *Platycodon grandiflorum* (Jacq.) exhibited comparable potent inhibitory effect of AGE (IC_50_ = 16.6 µM) with chlorogenic acid methyl ester (IC_50_ = 12.9 µM) [111].

#### 3.4.42. Luteolin-7-*O*-*β*-d-glucopyranoside

Molecular formula: C_21_H_20_O_11_ (488.38 g/mol), Melting point: >195 °C.

Luteolin-7-*O*-*β*-d-glucopyranoside was isolated from the leaves extract of *Stauntonia hexaphylla* (Thunb.) Decne. (stauntonia vine), traditionally used as folk medicine in China, Japan and Korea. Luteolin-7-*O*-*β*-d-glucopyranoside showed the highest potent inhibitory activity of RLAR at IC_50_ value of 7.34 µM among other isolated secondary metabolites. Inhibition was reported 2.4 times higher compared to that of quercetin. This data was substantiated by inhibition of AGE at IC_50_ value of 117.8 µM and it was suggested that the presence of sugar position in flavonoids enhances the activity [117].

#### 3.4.43. Magnoflorine

Molecular formula: C_20_H_24_NO_4_^+^ (342.41 g/mol).

Magnoflorine was isolated from *Tinospora cordifolia* (heart-leaved moonseed, guduchi, giloy) [118] and *Coptidis rhizome* (coptis root, huang lian) [88] for inhibitory activities against AR. Identification of magnoflorine was conducted with spectroscopic analysis and compared with the literature for both plants. Appeared as yellow powder, this compound exhibited lowest concentration of maximum RLAR activity showing IC_50_ value at 3.6 µM from isolation of *Tinospora cordifolia*. Further analysis showed that magnoflorine inhibited 72.3% of galactose-induced polyol accumulation [118]. Nevertheless, the isolated magnoflorine from *Coptidis rhizome* possessed marginal inhibition against RLAR with 18% inhibition at a concentration of 146 µM. At this point, it is not clear if the very significant differences in efficacies and potencies were due to technical differences in isolation and/or biological assay.

#### 3.4.44. Methyl-3,5-di-*O*-caffeoylquinate

Molecular formula: C_26_H_26_O_12_ (530.50 g/mol).

Methyl-3,5-di-*O*-caffeoylquinate or also known as 3,5-di-*O*-caffeoylquinic acid methyl ester was isolated from the flowers of *Erigeron annuus* [68] and fruits of *Xanthium strumarium* [33] with highest inhibitory activity towards RLAR among all isolated secondary metabolites. Phytochemical analysis of ethyl acetate-soluble fraction of *Erigeron annuus* methanolic flower extract afforded methyl-3,5-di-*O*-caffeoylquinate with yellow gum appearance at percentage yield of 0.0075% [68]. Isolated methyl-3,5-di-*O*-caffeoylquinate from both plants suppressed RLAR activity at IC_50_ values of 0.3 to 0.81 µM. Significant results (most potent) were also observed with further assays in RHAR and galactitol accumulation in rat lenses ex vivo from *Xanthium strumarium* at IC_50_ value of 0.67 µM and 117 µg/lens wet weight, respectively [33].

#### 3.4.45. Mumeic Acid-A

Molecular formula: C_24_H_24_O_10_ (472.44 g/mol).

In an attempt to obtain inhibitors of RLAR from *Prunus mume* (Japanese apricot), mumeic acid-A was found to be the most potent inhibitor from all isolated secondary metabolites. The IC_50_ concentration of mumeic acid-A (IC_50_ = 0.4 µM) was almost twice that of chlorogenic acid (IC_50_ = 0.7 µM) as the positive control [119,120].

#### 3.4.46. Puerariafuran

Molecular formula: C_16_H_12_O_5_ (360.32 g/mol), Melting point: 294–296 °C.

The roots of *Pueraria lobata* has for long been used in Far Eastern Asia countries as traditional medicine [57]. The isolation of root extract affords puerariafuran, a new 2-arylbenzofuran inhibited RLAR with an IC_50_ value of 22.2 µM, much lower than the positive control, 3,3-Tetramethyleneglutaric acid (IC_50_ = 28.8 µM). This data was substantiated with prevention of xylose-induced lens opacity in a dose-dependent manner, with the highest dose at 15 µM [121].

#### 3.4.47. Quercetin-3-*O*-*β*-d-glucoside

Molecular formula: C_21_H_20_O_12_ (464.38 g/mol).

Quercetin-3-*O-β*-d-glucoside was isolated from *Petasites japonicus* (butterbur, fuki, sweet coltsfoot) and *Stauntonia hexaphylla* as inhibitor for RLAR and AGE activities. It inhibited RLAR activity at IC_50_ values between 2.21 and 10.4 µM [117,122]. In contrast, its inhibition of AGE formation required a much higher concentration (IC_50_ = 1 mM) [117].

#### 3.4.48. Quercitrin (Quercetin 3-*O*-*α*-l-rhamnoside)

Molecular formula: C_21_H_20_O_11_ (448.38 g/mol), Melting point: 177–183 °C

Quercitrin, a glycosylation of quercetin at C-3, was isolated from few botanicals including *Smilax china* L. (China root), *Agrimonia pilosa* Ledeb, *Allium victorialis* var. *platyphyllum*, and *Melastoma sanguineum* using various isolation methods. It was tested for RLAR and AGE inhibitory assays and found that quercitrin possesses significant inhibitory actions for both activities among all isolated phytoconstituents in these plants. The IC_50_ values were reported at 0.17 to 0.56 µM (RLAR) and 42.0 to 58.0 µM (AGE) [99,100,123]. In contrast, quercitrin from *Melastoma sanguineum* showed IC_50_ value of 0.16 µM (RLAR) and 25.11 µM (AGE), respectively [40].

#### 3.4.49. Rhetsinine

Molecular formula: C_19_H_17_N_3_O_2_ (319.36 g/mol), Melting point: 196 °C.

*Evodia rutaecarpa* Bentham (Rutaceae) (wu zhu yu) is part of the Kampo-herbal medicine in Japan and has been used to relive digestion as well as painkiller. Various compounds have been isolated from this plant especially rhetsinine showed potent inhibitory activity against RLAR at an IC_50_ value of 24.1 µM. Rhetsinine was also reported to inhibit sorbitol accumulation in human erythrocyte by almost 79.3% at 100 µM [124].

#### 3.4.50. Rosmarinic Acid

Molecular formula: C_18_H_16_O_8_ (360.32 g/mol), Melting point: 171–175 °C.

Rosmarinic acid, a conjugation of caffeic acid and 3,4-dihydroxyphenyllactic acid is mainly isolated from the family of Lamiaceae with good anti-cataract activity. Isolated rosmarinic acid from *Salvia grandifolia* (da ye shu wei cao) recorded the lowest IC_50_ (0.30 µM) in RLAR activity [125] compared to other plants. The IC_50_ values of rosmarinic acid isolated from other plants were 2.77 µM (*Prunella vulgaris* L.; woundwort, self-heal) [70], 5.38 µM (*Colocasia esculenta* (L.) Schott; taro) [126], and 11.2 µM (seeds, *Perilla frutescens* L.) [113], respectively. In RHAR assay, the activity of rosmarinic acid were shown at 2.77 µM (leaves, *Perilla frutescens* L.) [69] and 18.6 µM (*Prunella vulgaris* L.) with potent activity on galactitol accumulation in rat lenses, but low inhibitor of AGE (20.7%) for *Prunella vulgaris* L. [70].

#### 3.4.51. Scopoletin

Molecular formula: C_10_H_8_O_4_ (192.16 g/mol), Melting point: 204–205 °C.

The bioassay-guided fractionation of methanol extract of *Magnolia fargesii* (Shin-i) air-dried buds yielded five compounds with scopoletin showing the most potent in RLAR, AGE and xylose-induced lens opacity assays [127]. Scopoletin significantly inhibits AGE formation with an IC_50_ value of 2.9 µM, approximately 327 times more potent compared to the positive control (IC_50_ = 961 µM). Similarly, in the RLAR activity, scopoletin showed marked inhibitory activity with an IC_50_ value of 22.5 µM. This was substantiated by the suppression of lens opacity to 72.9% (25 µM) after three days of xylose treatment [127]. A lower IC_50_ concentration of RLAR activity was observed for scopoletin isolated from *Angelica gigas* (dang gui, Korean angelica) with an IC_50_ value of 2.6 µM [128] showing the most potent activity among all isolated secondary metabolites. However, scopoletin from methanolic young leaves of *Artemisia montana* showed higher IC_50_ value at 64.5 µM for the same activity [52,129].

#### 3.4.52. Semilicoisoflavone B

Molecular formula: C_20_H_16_O_6_ (352.34 g/mol), Melting point: 131–134 °C.

Semilicoisoflavone B is mostly found in roots and rhizomes of licorice species (*Glycyrrhiza* sp.) [130]. In searching for potential AR inhibitors, 10 secondary metabolites have been isolated from bioactivity-guided isolation of *Glycyrrhiza uralensis* with semilicoisoflavone B showed the most potent inhibition of RLAR and RHAR activities. Both inhibition rates were recorded at IC_50_ values of 1.8 and 10.6 µM, respectively. Unlike γ,γ-dimethylallyl type prenylated isoflavonoids, semilicoisoflavone B containing γ,γ-dimethylchromene ring on the aromatic ring inhibited AR more strongly. In kinetic analysis of AR inhibition, semilicoisoflavone B did not bind to any substrate and NADPH binding regions of RHAR. Ex vivo analysis showed that this compound highly inhibited sorbitol accumulation in rat lenses incubated with high glucose by 47.0% [131].

#### 3.4.53. Sulfuretin and Butein

Molecular formula of sulfuretin: C_15_H_10_O_5_ (270.24 g/mol), Melting point: 295–303 °C.

Molecular formula of butein: C_15_H_12_O_5_ (272.25 g/mol), Melting point: 216 °C.

The AR and AGE guided isolation of ethanolic bark extract of *Rhus verniciflua* (lacquer tree) produced nine secondary metabolites with sulferetin and butein as the most potent phytoconstituents for AGE and RHAR, respectively. Sulferetin was isolated as white to off-white crystalline powder [132] and inhibited against AGE activity at IC_50_ value of 124 µM, 11 times lower than aminoguanidine (IC_50_ = 1450 µM). The RHAR inhibitory activity of butein was reported at IC_50_ = 0.7 µM [133]. The efficacies of both phytoconstituents have been suggested on the structure activity relationships of catechol moiety of the B ring and 4′-hydroxyl at the A ring for butein [134] and hydroxyl groups of flavones at the 3′-, 4′-, 5′-, and 7-positions for sulferetin [135].

#### 3.4.54. Syringic Acid

Molecular formula: C_9_H_10_O_5_ (198.17 g/mol), Melting point: 206–208 °C.

Syringic acid is a phenolic compound and a naturally occurring *O*-methylated trihydroxybenzoic acid monomer extracted from *Herba dendrobii* (shi hu)*. Herba dendrobii*, found in the stem of many orchid species of the *Dendrobium* genus, has been used to improve vision centuries ago [136]. Syringic acid at medium dose (79.97%) isolated from *Herba dendrobii* improves survival of high-concentration d-galactose-injured human LEC with inhibition ratio of 20.3%. Rat lens turned clear to Grade 0 after 90 days of treatment. Syringic acid inhibits AR activity in a dose-dependent manner with an IC_50_ value of 213.17 µg/mL (1075.7 µM). Data suggest that syringic acid downregulates the expression of mRNA of AR [136]. However, the AR inhibition by syringic acid isolated from *Magnolia officinalis* was weaker with less than 10% of inhibition [137].

#### 3.4.55. Swertisin

Molecular formula: C_22_H_22_O_10_ (446.40 g/mol), Melting point: 243 °C.

Swertisin appears as pale yellow powdery crystals and isolated from *Enicostemma hyssopifolium* (najajihva, chota chirayita) methanol extract after repeated column chromatography over silica gel. This compound reacts with ferric chloride and turned greenish brown color as a confirmation test for flavonoids. RLAR activity was significantly inhibited by swertisin at an IC_50_ value of 0.71 µg/mL (1.6 µM; 82.3% inhibition at 10 µg/mL) indicating a higher inhibition compared to the other compound isolated, swertiamarin (IC_50_ = 7.59 µg/mL). This compound was also found to suppress polyol accumulation (41.7%) in lenses cultured in a galactitol medium [138].

#### 3.4.56. Valoneic Acid Dilactone

Molecular formula: C_21_H_10_O_13_ (470.29 g/mol), Melting point: 177–183°C.

The repeated column chromatography and preparative HPLC of seed methanolic extract of *Syzygium cumini* (L.) Skeels lead to the isolation of six phytoconstituents with valoneic acid dilactone showed the highest activity against RLAR inhibitory activity at IC_50_ value of 0.075 µM [87]. Valoneic acid dilactone were the first constituents from this plant reported to possess RLAR inhibitory activity.

## 4. Discussion and Outlook

Despite the success in surgical replacement of the cataractous lens with an artificial intraocular lens, discovery of pharmacological prevention and treatment of this blinding disorder has been an earnest, continuous effort in ophthalmology research. In this review manuscript, we summarize findings of phytoconstitutents and their pharmacological effects as potential anti-cataract agents. The large number of interesting compounds is exciting. It raises hope that clinically useful medication may have a good chance to be derived from this sizable collection of chemicals with diverse structural scaffolds.

Many of the compounds have potent and efficacious in vitro pharmacological activities that are consistent with potential anti-cataract effects. For example, 1,2,3,6-tetra-*O*-galloyl-*β*-d-glucose inhibits AGE formation with an IC_50_ of 2 M. Both 1,3,5,8-tetrahydroxyxanthone and 2′′,4′′-*O*-diacetylquercitrin inhibit AR with IC_50_ values below 0.1 M. However, a major limitation of the listed compounds is that although they have been shown to have the appropriate biological actions in a variety of in vitro or ex vivo assays, many of them were not tested in animal cataract models. Additionally, a few have been evaluated in only one animal model. Without relevant in vivo data, it is obviously very difficult to develop the compounds into meaningful treatments for cataract patients.

In addition to the lack of in vivo data, there are other challenges facing development of anti-cataract pharmaceuticals. For example, cataract medication has to compete with the very successfully and generally affordable (as least in developed countries) surgical procedure. Moreover, pharmacological prevention of cataract formation is expected to require a long-term, likely multi-year, administration of medicine, which, to some, is undesirable. Overcoming these challenges necessitates careful considerations of drug safety, convenience of administration, and cost. These concerns may have previously prohibited the development of certain agents. Nonetheless, we feel that phytoconstituents are advantageous compared to conventional synthetic drugs. Many societies have been using plant products from where some of the ingredients are derived for centuries, indicating long-term safety and acceptance. The development path and clinical use will be similar to vitamins and phytochemicals such as lutein and zeaxanthine. If proven safe, cost-effective, and most importantly, efficacious in preventing or reversing cataract formation, phytoconstituents can be a revolutionary approach in the treatment of cataract.

## 5. Conclusions

Despite the success in surgical replacement of the cataractous lens with artificial intraocular lens, pharmacological prevention and treatment of this blinding disorder have been an earnest, continuous effort in ophthalmology research. In this review manuscript, we summarize findings of 56 entries of phytoconstitutents and their pharmacological effects as potential anti-cataract agents. The large number of interesting compounds is exciting. It raises hope that clinically useful medication may have a good chance to be derived from this sizable collection of chemicals with diverse structural scaffolds.

Many of the compounds have potent and efficacious in vitro pharmacological activities that are consistent with potential anti-cataract effects. For example, 1,2,3,6-tetra-*O*-galloyl-*β*-d-glucose inhibits AGE formation with an IC_50_ of 2 µM. Additionally, both 1,3,5,8-tetrahydroxyxanthone and 2′′,4′′-*O*-diacetylquercitrin inhibit AR with IC_50_ values below 0.1 µM. However, a major limitation of the listed compounds is that although they have been shown to have the appropriate biological actions in a variety of in vitro or ex vivo assays, many of them were not tested in animal cataract models. And a few have been evaluated in only one animal model. Without relevant in vivo data, it is obviously very difficult to develop the compounds into meaningful treatments for cataract patients. We feel that, by listing the comprehensive collection of phytocontituents in one place, this manuscript serves as an overview and perhaps an inspiration to prompt additional studies in this important research area. Collaborative efforts between phytochemists and cataract researchers are promisingly fruitful.

In addition to the lack of in vivo data, there are other challenges facing development of anti-cataract pharmaceuticals. For example, cataract medication has to compete with the very successfully and generally affordable (as least in developed countries) surgical procedure. Moreover, pharmacological prevention of cataract formation is expected to require a long-term, likely multi-year, administration of medicine, which, to some, is undesirable. Overcoming these challenges necessitates careful considerations of drug safety, convenience of administration, and cost. These concerns may have previously prohibited the development of certain agents. Nonetheless, we feel that phytoconstituents are advantageous compared to conventional synthetic drugs. Many societies have been using plant products where some of the ingredients are derived from for centuries, indicating long-term safety and acceptance. The development path and clinical use will be similar to vitamins and phytochemicals such as lutein and zeaxanthine. If proven safe, cost-effective, and most importantly, efficacious in preventing or reversing cataract formation, phytoconstituents can be a revolutionary approach in the treatment of cataract.

## Figures and Tables

**Table 1:** Summary of relevant in vitro anti-cataract activities of phytoconstituents.

Active Ingredient	Structure	IC_50_ Values						
		AGE	ARI	GLWW	RHAR	BLAR	HLAR	RLAR
1-*O*-galloyl-*β*-d-glucose (β-Glucogallin)	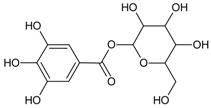	NA	NA	NA	17.00 µM [30]	NA	NA	NA
1,3-di-*O*-caffeoylquinic acid	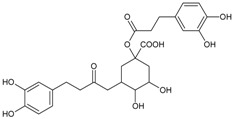	24.85 µM [32]	NA	NA	0.810 µM [33]	NA	NA	0.22 µM [32]
1,5-Di-hydroxy-1,5-di-[(*E*)-3-(4-hydroxyphenyl)-2-propenoic]-3-pentanonyl ester (DHDP)	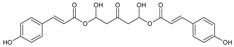	NA	NA	NA	194.67µM [34]	NA	NA	NA
1,5-di-*O*-caffeoylquinic acid	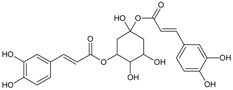	NA	NA	NA	NA	NA	NA	2.98 µM [35]
1,3,6-trihydroxy-2-methoxymethylanthraquinone	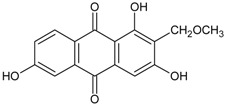	52.72 µM [36]	NA	NA	NA	NA	NA	3.04 µM [36]
1,2,3,6-tetra-*O*-galloyl-*β*-d-glucose	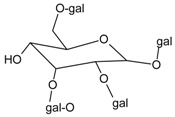	1.99 µM [38]	NA	NA	NA	NA	NA	0.70 µM [38]
1,3,5,8-Tetrahydroxyxanthone	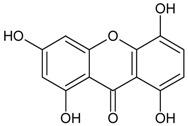	NA	NA	NA	NA	NA	NA	0.0886 μM [39]
2″,4″-*O*-Diacetylquercitrin	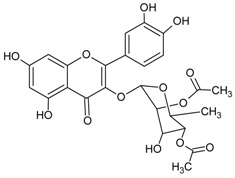	11.46 µM [40]	NA	NA	NA	NA	NA	0.077 µM [40]
3-Isomangostin	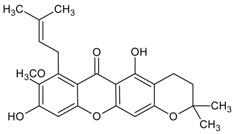	NA	NA	NA	NA	NA	NA	3.48 μM [41]
3′,5′-dimethoxy-(1,1′-biphenyl)-3,4-diol 3-*O*-*β*-d-glucopyranoside	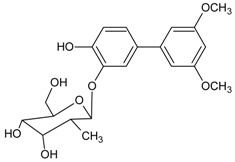	NA	NA	NA	NA	NA	NA	3.80 µM [47]
3,5-di-*O*-caffeoylquinic Acid	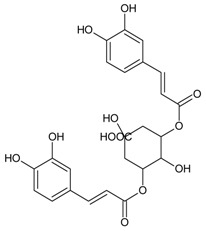	6.06 µM [48]	NA	153 g [33]	1.34 µM [33]	NA	NA	0.19 µM [33]
4-*O*-butylpaeoniflorin	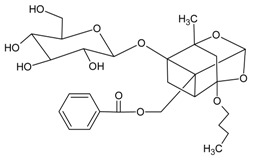	10.80 µM [53]	NA	NA	NA	NA	NA	36.20 µM [53]
4,5-Di-*O*-trans-caffeoyl-d-quinic acid	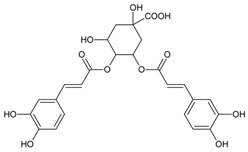	NA	NA	NA	NA	NA	NA	0.29 µM [55]
5-*O*-Feruloly quinic acid	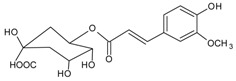	NA	NA	NA	NA	NA	NA	14.19 µM [56]
5,7,4′-trihydroxyisoflavone (Genistein)	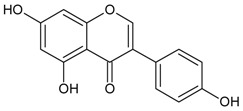	NA	NA	NA	NA	NA	NA	9.48 µM [60]
20(*S*)-Ginsenoside Rh2	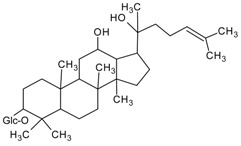	NA	NA	NA	147.40 µM [61]	NA	NA	NA
Acteoside	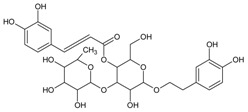	5.11 µM [63]	NA	NA	NA	NA	NA	0.83 µM [63]
Basilicumin [7-(3-hydroxypropyl)-3-methyl-8-*β*-*O*-d-glucoside-2H-chromen-2-one]	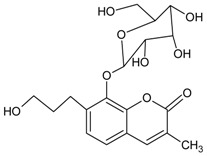	NA	NA	NA	NA	2.09 µM	NA	NA
Caffeic acid	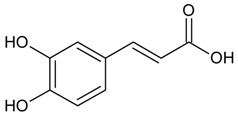	7.56 µM [68]	NA	NA	210.28µM [66]	NA	NA	16.71 µM [65]
Canangafruiticoside E	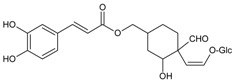 Glc=β-d-glucopyranoside	NA	NA	NA	NA	NA	NA	0.80 µM [71]
Capsofulvesin A [((2*S*)-l-*O*-(6Z,9Z,12Z,15Zoctadecatetraenoyl)-2-*O*-(4Z,10Z,13Zhexadecatetraenoyl)-3-*O*-*β*-d-galactopyranosyl glycerol)]	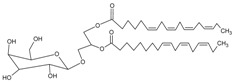	NA	NA	NA	NA	NA	NA	52.53 µM [72]
Davallialactone	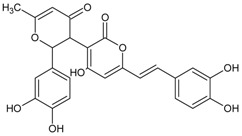	NA	NA	NA	0.56 µM [67]	NA	NA	0.33 µM [67]
Delphinidin 3-*O*-*β*-galactopyranoside-3′-*O*-*β*-glucopyranoside	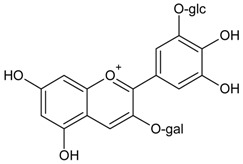 Glc= β-glucopyranoside, Gal= β-galactopyranoside	NA	NA	NA	NA	NA	NA	0.37 µM [83]
Desmethylanhydroicaritin	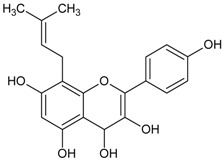	294.60 µM [84]	NA	NA	0.45 µM [84]	NA	NA	0.95 µM [84]
Ellagic acid	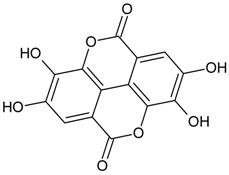	26.0 µM [86]	NA	42.47% [85]	NA	NA	1.37 µM [67]	0.12 µM [87]
Epiberberine	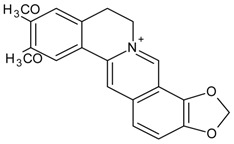	NA	NA	NA	168.10 µM [88]	NA	NA	100.07 µM [88]
Geraniin	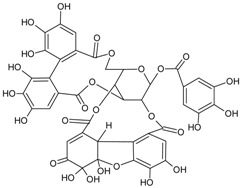	21.00 µM 96% * [89]	0.15 µM [89]	39.87% [85]	NA	NA	NA	NA
Hipolon	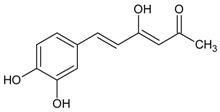	NA	NA	NA	NA	NA	NA	9.47 µM [90]
Hirsutrin	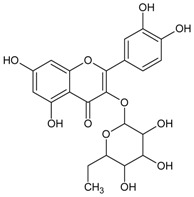	NA	NA	33.78% [91]	NA	NA	NA	4.78 µM [91]
Hopeafuran	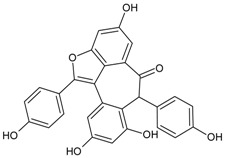	NA	NA	NA	NA	NA	NA	14.80 µM [92]
Hypolaetin 7-*O*-[6′′′-*O*-acetyl-*β*-d-allopyranosyl-(1→2)]-6′′-*O*-acetyl-*β*-d-glucopyranoside	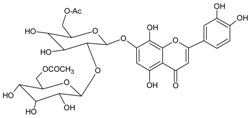	NA	0.66 µM [93]	NA	NA	NA	NA	NA
Isocampneoside II	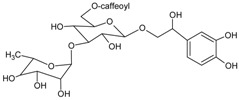	NA	NA	NA	9.72 µM [66]	NA	NA	NA
Kaempferol	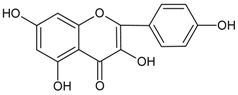	36.01 µM [100]	NA	NA	45.58 µM [66]	NA	NA	1.10 µM [98,100]
Kakkalide	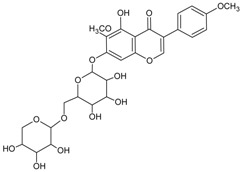	NA	NA	NA	NA	NA	NA	0.56 µM [101]
Lucidumol A [(24*S*)-24,25-Dihydroxylanost-8-ene-3,7-dione]	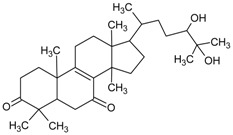	NA	NA	NA	NA	19.10 µM [102]	NA	NA
Lupeol	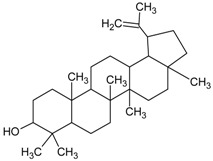	NA	NA	NA	3.60 µM [107]	NA	NA	NA
Luteolin (2-(3,4-dihydroxyphenyl)-5,7-dihydroxy-4-chromomenone)	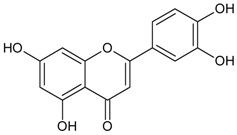	16.60 µM [111]	NA	NA	6.34 µM [52]	NA	NA	0.087 µM [111]
Luteolin-7-*O*-*β*-d-glucopyranoside	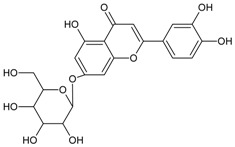	117.80 µM [117]	NA	NA	NA	NA	NA	7.34 µM [117]
Magnoflorine	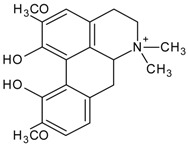	NA	NA	NA	NA	NA	NA	3.60 µM [118]
Methyl-3,5-di-*O*-caffeoylquinate	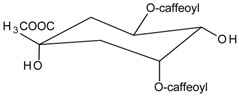	NA	NA	117 g [33]	0.67 µM [33]	NA	NA	0.30 µM [33]
Mumeic acid-A	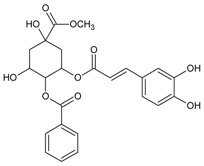	NA	NA	NA	NA	NA	NA	0.40 µM [119]
Palbinone	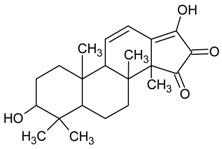	>500 µM [53]	NA	NA	NA	NA	NA	11.40 µM [53]
Puerariafuran	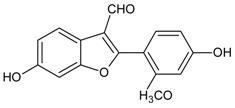	NA	NA	NA	NA	NA	NA	22.20 µM [57,121]
Quercetin-3-*O*-*β*-d-glucoside	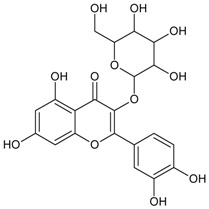	>1000 µM [117]	NA	NA	NA	NA	NA	2.21 µM [122]
Quercitrin (Quercetin 3-*O*-*α*-l-rhamnoside)	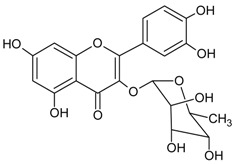	4.20 µM [100]	NA	NA	NA	NA	NA	0.17 µM [40]
Rhetsinine	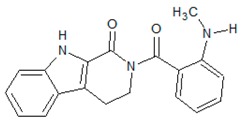	NA	NA	NA	NA	NA	NA	24.10 µM [124]
Rosmarinic acid	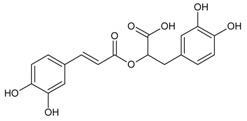	NA	NA	532.38g [70]	2.77 µM [69]	NA	NA	0.30 µM [125]
Scopoletin	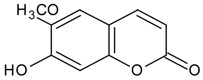	2.93 µM [127]	NA	NA	NA	NA	NA	2.59 µM [128]
Semilicoisoflavone B	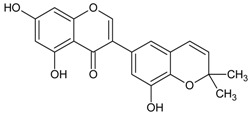	NA	NA	NA	10.60 µM [131]	NA	NA	1.80 µM [131]
Sulfuretin	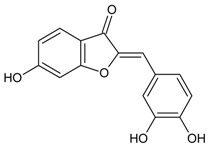	124.00 µM [133]	NA	NA	1.30 µM [133]	NA	NA	NA
Syringic Acid	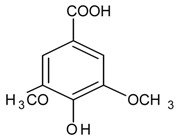	NA	NA	NA	NA	NA	NA	1081.1 µm [136]
Swertisin (C-glycosidic flavonoid)	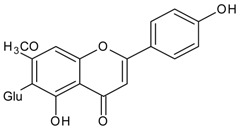 Glu=glucose	NA	NA	NA	NA	NA	NA	1.60 µm [138]
Valoneic acid dilactone	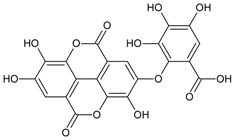	NA	NA	NA	NA	NA	NA	0.075 µM [87]

**Table 2:** Summary of relevant ex vivo and in vivo activities of phytoconstituents.

Constituent Name (Class of Constituent)	Structure	Doses (IC_50_/EC_50_)					
		AR Transgenic Mice	Selenite-Induced	AR Rat Lens	Galactose-Induced Lens Opacity	Xylose-Induced Lens Opacity	Ref
1-*O*-galloyl-*β*-d-glucose (β-Glucogallin)	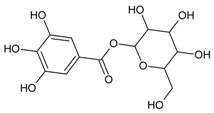	Ex vivo: 30.00 µM	NA	NA	NA	NA	[29]
1,2,3,6-Tetra-*O*-galloyl-*β*-d-glucose	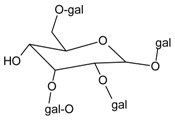	NA	NA	NA	NA	Ex vivo: 80.00 µM	[38]
3,5-di-*O*-caffeoyl-epi-quinic Acid	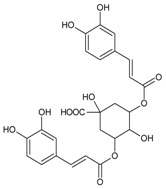	NA	NA	NA	NA	Ex vivo: 10.00 μM	[48]
5,7,4′-trihydroxyisoflavone (Genistein)	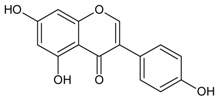	NA	NA	NA	NA	Ex vivo: 18.50 µM	[60]
Isorhamnetin-3-glucoside	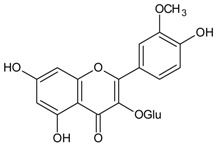	NA	Ex vivo: 52.25 µM	NA	NA	NA	[97]
Lupeol	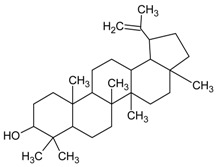	NA	In vivo: 126.15 μM	NA	NA	NA	[108]
Luteolin (2-(3,4-dihydroxyphenyl)-5,7-dihydroxy-4-chromomenone)	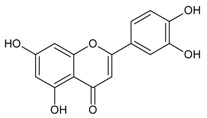	NA	Ex vivo: 6.98 µM	NA	NA	NA	[112]
Puerariafuran	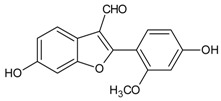	NA	NA	NA	NA	Ex vivo: 15.00 µM	[121]
Scopoletin	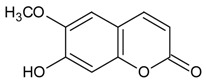	NA	NA	NA	NA	Ex vivo: 25.00 µM	[127]
Syringic acid	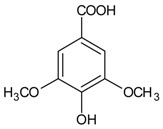	NA	NA	NA	Ex vivo: 1075.70 μM In vivo: 2% syringic acid eye drop (131,197.80 μM)	NA	[136]

## Abbreviations

AGE	Advanced glycation end-product
AR	Aldose reductase
ARI	Aldose reductase inhibition
BLAR	Bovine lens aldose reductase
GC	Glucocorticoid
GSH	Glutathione
HLAR	Human lens aldose reductase
LEC	Lens epithelial cell
NADPH	Nicotinamide adenine dinucleotide phosphate
RHAR	Recombinant human aldose reductase
RLAR	Rat lens aldose reductase
ROS	Reactive oxygen species
SDH	Sorbitol dehydrogenase
STZ	Streptozotocin
UV	Ultraviolet
